# Plant Organellar Proteomics in Response to Dehydration: Turning Protein Repertoire into Insights

**DOI:** 10.3389/fpls.2016.00460

**Published:** 2016-04-13

**Authors:** Deepti B. Gupta, Yogita Rai, Saurabh Gayali, Subhra Chakraborty, Niranjan Chakraborty

**Affiliations:** ^1^Department of Biotechnology, TERI UniversityNew Delhi, India; ^2^National Institute of Plant Genome Research, Jawaharlal Nehru University CampusNew Delhi, India

**Keywords:** adaptive responses, crop yield, dehydration, subcellular proteome, stress signals, spatiotemporal regulation

## Abstract

Stress adaptation or tolerance in plants is a complex phenomenon involving changes in physiological and metabolic processes. Plants must develop elaborate networks of defense mechanisms, and adapt to and survive for sustainable agriculture. Water-deficit or dehydration is the most critical environmental factor that plants are exposed to during their life cycle, which influences geographical distribution and productivity of many crop species. The cellular responses to dehydration are orchestrated by a series of multidirectional relays of biochemical events at organelle level. The new challenge is to dissect the underlying mechanisms controlling the perception of stress signals and their transmission to cellular machinery for activation of adaptive responses. The completeness of current descriptions of spatial distribution of proteins, the relevance of subcellular locations in diverse functional processes, and the changes of protein abundance in response to dehydration hold the key to understanding how plants cope with such stress conditions. During past decades, organellar proteomics has proved to be useful not only for deciphering reprograming of plant responses to dehydration, but also to dissect stress–responsive pathways. This review summarizes a range of organellar proteomics investigations under dehydration to gain a holistic view of plant responses to water-deficit conditions, which may facilitate future efforts to develop genetically engineered crops for better adaptation.

## Introduction

Agricultural productivity and food security is subject to increasing environmental constraints, particularly to water-deficit condition due to its high magnitude of damage and global impact (Bartels and Sunkar, 2005). One-third of arable land worldwide suffers from chronic or at least transient water scarcity, which is directly proportional to the reduction of crop yield. It is estimated that the world population will be about 8.5 billion by 2030, an increase of 22% from the current population, compelling an imminent rise in food demand. Additionally, the altered precipitation patterns, onset by unpredictable changes in climate, are speculated only to worsen, which is a major threat to global food security (Boyer, 1982). Hence, understanding how plants respond to water-deficit is the key factor for developing strategies for crop improvement.

Most environmental stresses are characterized by unifying feature that, at least a part of their detrimental effect on plant performance, is caused by unavailability of water. As a convergent point of multiple abiotic cues, cellular effects of dehydration stress are not only imbalances of ionic and osmotic homeostasis, but also impaired photosynthesis and cellular energy depletion, besides oxidative damage to the cellular machinery (Verslues et al., 2006). The current challenge is to identify elements involved in perception of stress and its path of translation in the cellular machinery to induce adaptive response. The present understanding of stress signaling in plants is largely based on genomic studies, which have been postulated upon comparative modulation of gene expression in response to stress (Seki et al., 2002; Shinozaki et al., 2003; Golldack et al., 2014). However, dehydration stress response is a complex multigenic phenomenon, where transcriptome expression rarely translates in equivalence with functional proteomic signatures (Gygi et al., 1999; Bohnert et al., 2006). Increasing evidence suggest a synergistic relationship between quantitative and qualitative changes in dehydration-induced proteins and physiological adaptations of plants (Bhushan et al., 2007; Pandey et al., 2010; Chen et al., 2011). The dehydration-responsive changes in the proteome can be grouped into five categories: (1) accumulation of dehydration-responsive proteins, which are characteristics of stress defense (Bartels and Sunkar, 2005; Mittler, 2006); (2) reorganization of the proteome profile among the organelles to optimize resources and achieve cellular homeostasis (Vera-Estrella, 2005; Poschet et al., 2011); (3) association network of multi-functional proteins linked to altered spatial distribution (Wu et al., 2009; Pandey et al., 2010); (4) PTM of regulatory proteins leading to activation or deactivation of metabolic pathways or isoform variants (Lata et al., 2011); and (5) reprograming of species-specific metabolic pathways, typically involved in stress tolerance (Bhushan et al., 2006; Pandey et al., 2010; Jaiswal et al., 2012). Finally, emerging evidence suggest that the phenotypic plasticity of plants, which limits stress-induced damage, is a direct result of altered proteome dynamics at the organellar level. A clear understanding of all these functional modulations can only be achieved by systematic analysis of dehydration-induced subcellular proteomes and unwinding their elaborate interaction networks.

During the past decade, a number of stress-responsive organellar proteomics studies have been carried out on variety of plant species, and increasingly agriculturally important crop species are being investigated against model plants (Pandey et al., 2008; Komatsu et al., 2009; Bhushan et al., 2011; Jaiswal et al., 2012; Hossain and Komatsu, 2013). This is primarily due to the availability of different genotypes of a crop with variable degrees of stress tolerance. Hence, comparative analysis of contrasting genotypes is a targeted approach for selectively identifying elements conferring relative tolerance against dehydration-induced damages (Bhushan et al., 2006; Pandey et al., 2008; Komatsu et al., 2009; Jaiswal et al., 2012; Verma et al., 2014). Even though numerous studies have been carried out on the plant stress response, a deeper insight into dehydration response at the sub-proteomic level is far from complete. This review will highlight the role of each subcellular compartment in sequence of events such as perception of dehydration stress, signal relay, and finally their cooperative function to combat stress. It will also critically discuss the altered dynamics of organelle crosstalk under dehydration and attempt to build a comprehensive spatiotemporal regulation of events that drive signaling pathways in plants under water-deficit.

## Dehydration-Responsive Cell Wall Proteome

**FIGURE 1:**
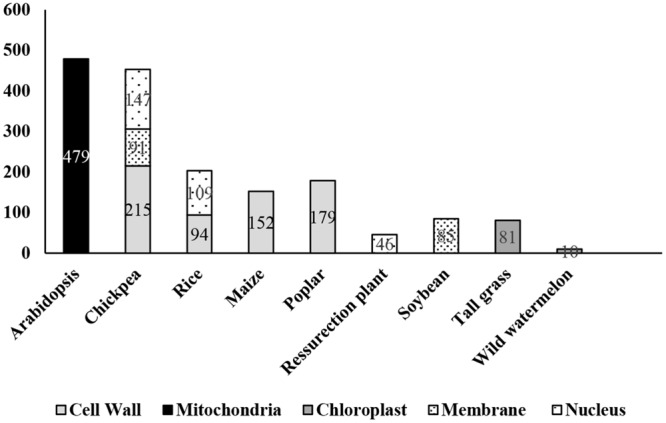
**Number of dehydration-responsive proteins identified in different plant species.** Numerical within the bar represents proteins reported from different proteomics studies.

Plant cell wall or ECM acts as a front-line defense, and is a conduit for signal transduction between the apoplast and the symplast, thereby plays a key role in cell fate decision under dehydration stress. Although proteins account for only 10% of the ECM mass, they comprise several hundreds of different molecules with diverse cellular functions (Carpita and Gibeaut, 1993). A detailed study of dehydration-responsive ECM proteome of chickpea identified 134 DRPs (Bhushan et al., 2007) and over 100 DRPs in rice (Pandey et al., 2010), presumably involved in an array of cellular functions. A similar study in maize primary root elongation zone led to the identification of water-soluble and loosely ionically bound 152 DRPs in the cell wall (Zhu et al., 2007). Pechanova et al. (2010) examined the apoplast of poplar stem and leaf tissues and reported 247 differentially regulated proteins (**Table 1**, **Figure 1**). The leaf apoplast showed abundance of proteins involved in cell wall metabolism, while the stem apoplast comprised of proteins mostly associated with cell defense. Despite the variation, the differential cell wall proteomes across the crop species showed commonalities in the essential functional protein classes viz., defense response, particularly ROS management and cell wall modification, albeit many proteins were observed to be unique to each of the crops studied. Cell wall, as emerged from these studies, is a repository of signaling molecules suggesting that the communication between the ECM and the symplast is one of the characteristic features of cellular mechanism that allows cells to respond effectively to various extracellular signals. Multivariate protein signatures such as WAKs, protein kinases, NDK, inorganic phosphatases and GTPases, among others were identified from both rice and chickpea seedlings (Bhushan et al., 2007; Pandey et al., 2010). The WAKs and protein kinases are known to relay extracellular signals through their cytoplasmic kinase domain and bind to a 2C-type protein phosphatase in the cytoplasm to form a signalosome complex (Anderson et al., 2001). NDKs identified both in rice and chickpea interact with cytosolic catalases and play a key role in relieving oxidative stress (Fukamatsu et al., 2003). Furthermore, 14-3-3 superfamily proteins were identified in the rice cell wall (Pandey et al., 2010), which have a characteristic role in stress signaling via modulating ROS accumulation (Elmayan et al., 2007). Another mechanism established for stress perception and signaling under dehydration is by induction, due to stretch-activated cytoskeletal channels. Several key proteins such as mannose lectin and aldolases were identified, which participate in compensatory mechanisms of cytoskeletal rearrangement under stress (Jewett and Sibley, 2003; Garaeva et al., 2006). Interestingly, deregulation of signaling proteins were also observed in maize and poplar tissues (Pechanova et al., 2010).

**Table 1:** Large-scale subcellular proteome studies under water-deficit conditions.

Cell fraction	Plant	Number of proteins	Tissue	Proteomic method	Reference
Cell wall/Apoplast/ECM	Chickpea	134	Leaf	2-DE, LC-ESI-MS/MS	Bhushan et al., 2007
	Chickpea	81	Leaf	2-DE, LC-ESI-MS/MS	Bhushan et al., 2011
	Rice	94	Leaf	2-DE, LC-ESI-MS/MS	Pandey et al., 2010
	Maize	152	Root elongation zone	2-DE, ESI, Q-TOF-MS/MS	Zhu et al., 2007
	Poplar	279	Leaf, Steam	2-DE, LC-MS/MS	Pechanova et al., 2010
Nuclear	Chickpea	147	Leaf	2-DE, LC/MS/TOF	Pandey et al., 2008
	Rice	109	Leaf	2-DE, LC/MS/TOF	Choudhary et al., 2009
	Resurrection plant	18	Leaf	2-D, MS/MS	Abdalla et al., 2010
	Resurrection plant	28	Leaf	i-TRAQ together with 2 DLC & ESI-MS/MS	Abdalla and Rafudeen, 2012
Membrane/PM	Soybean	85	Seedling	2-DE, nano-LC-MS/MS	Nouri and Komatsu, 2010
	Chickpea	91		2-DE, LC-ESI-MS/MS	Jaiswal et al., 2012
Mitochondria	*Arabidopsis*	62	Leaf	2-DE, Q-TOF-MS/MS	Taylor et al., 2009
	*Arabidopsis*	417	Leaf	^15^N labeling	Skirycz et al., 2011
Chloroplast	*Festuca arundinacea*	81	Leaf	Q-TOF -MS/MS	Kosmala et al., 2012
	Wild watermelon	60	Leaf	LC-MS/MS	Sanda et al., 2011

The apoplast is known to be a source of ROS production (Zhu et al., 2007; Moschou et al., 2008). Dehydration-induced ROS can act as signaling molecules for stress response, but above a certain threshold they lead to oxidative injury to many cellular components. Most of the dehydration tolerance mechanisms rely primarily on protection of the cellular structure wherein an important method is the control of the level of ROS or the limitation of damage caused by ROS. It is well established that dehydration increases ROS levels, particularly O_2_^-^ and H_2_O_2_ (Apel and Hirt, 2004; Papadakis and Roubelakis-Angelakis, 2005). Accumulation of apoplastic ROS can be viewed by *in situ* imaging with fluorescent indicator (Zhu et al., 2007). A critical screening of the cell wall proteome displayed several of the DRPs linked to antioxidative/detoxifying reactions, for example, APX, SOD, malate dehydrogenase, GPX, MDAR, DHAR, germin like protein, and oxalate oxidase, most of which showed an induced expression (Bhushan et al., 2007; Zhu et al., 2007; Pandey et al., 2010; Pechanova et al., 2010). Molecular chaperones and other proteins were also reported to be involved in the protection of cellular machinery. A novel phytoferritin, classically known as an iron storage protein, was identified in the cell wall of chickpea, which was postulated to have a significant role in ROS neutralization (Bhushan et al., 2007).

It is well established that under osmotic stress, cell wall serves as a source of sugars to maintain osmotic balance and undergoes lignification to avoid further water loss. Multiple stress-responsive proteins such as Ado-met, methyl transferases, AdoHcyase, adenosine kinase were identified (Bhushan et al., 2007; Zhu et al., 2007; Pandey et al., 2010; Pechanova et al., 2010), which are part of lignification pathway. Additionally, proteins such as cellulose synthase, beta galactosidases, xyloglucan hydrolase and hexosaminidase, and polygalacturonase that are known to utilize cell wall polysaccharides as an alternate carbon source under sugar depletion were also identified (Bhushan et al., 2007; Zhu et al., 2007; Pandey et al., 2010; Pechanova et al., 2010). Further, a better understanding of dehydration tolerance was built by a comparative cell wall proteomics study of tolerant and susceptible varieties of chickpea (Bhushan et al., 2011). The dehydration-responsive proteomes revealed that early perception, advanced signaling, notably cell wall restructuring, enhanced osmotic adjustment and better management of ROS are the keys to enhanced adaptation in plants.

## Dehydration-Responsive Nuclear Proteome

Nucleus senses and physiologically responds to stress via multimodal signaling pathways, which are combinations of multiple input cues attributed by various organelles. The consequence is signal-specific response often resulting in cascade of downstream signals leading to activation of sub-responses such as hormonal modulations, systemic actions, and secondary regulations (Narula et al., 2013). In recent years, there have been several reports on the changes in nuclear proteome in varied cellular events (Bae et al., 2003; Lee et al., 2006; Salzano et al., 2006; Henrich et al., 2007; Buhr et al., 2008; Pandey et al., 2008; Repetto et al., 2008, 2012; Choudhary et al., 2009; Abdalla et al., 2010; Cooper et al., 2011; Varma and Mishra, 2011; Abdalla and Rafudeen, 2012). There have been at least four reports on dehydration-responsive nuclear proteome from three crops, one each from chickpea (Pandey et al., 2008) and rice (Choudhary et al., 2009), and two from the resurrection plant *Xerophyta viscosa*, (Abdalla et al., 2010; Abdalla and Rafudeen, 2012). The study in chickpea (Pandey et al., 2008) identified 147 DRNPs putatively involved in diverse functions, predominantly gene regulation and transcription, cell defense, protein degradation and chromatin remodeling, while a similar study in rice (Choudhary et al., 2009) led to the identification of over 100 DRNPs, categorized in comparable classes (**Table 1**, **Figure 1**). The DRNPs have also been elucidated in resurrection plant, capable of surviving at 5% RWC for prolonged periods. The study focussed on understanding the late protection mechanisms by comparative analysis between plants at 35% RWC vs. fully hydrated plants.

**FIGURE 2:**
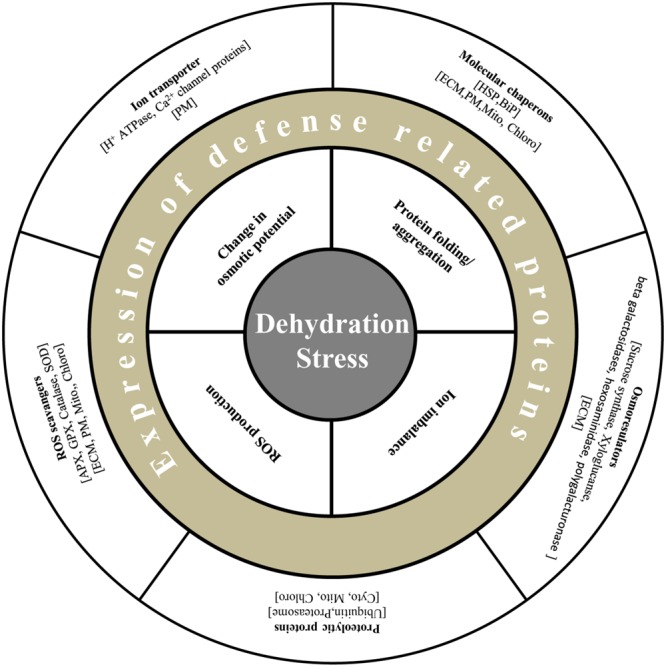
**Diagrammatic representation of defense related proteins under dehydration stress in plants.** Plants are able to alleviate dehydration stress by maintenance of turgor by osmoregulators such as sucrose synthase, xyloglucan, and exchange of ions by transporters such as H^+^ antiporters and ATPases. Generation of ROS is restricted by enzymes viz., APX, GPX, and SOD. Molecular chaperons like HSP and PIP maintain protein structure, and proteolytic enzymes degrade misfolded proteins.

A comparison of the studies mentioned above revealed intriguing facts on regulatory mechanisms controlling the dehydration response (**Figure 2**). The proteins involved in signaling and gene regulation were prominent, which comprised of transcription factors, nuclear trafficking proteins, protein kinases and phosphatases. The altered expression of well-characterized dehydration-responsive transcription factors such as WRKY, bZIP, and AP2 domain containing proteins was noticeable in rice and chickpea, but not in resurrection plant, probably because the study recorded late response. Signaling partners like protein kinase, serine–threonine kinase, histidine kinase, receptor like protein kinase, and tyrosine phosphatase were identified in rice and chickpea, while casein kinase was reported in resurrection plant. Proteins involved in nucleocytoplasmic transport such as RAN, RANbp, RAN GTPase WIP1, GTPase binding, and dynamin like proteins were found to be consistently up-regulated. These proteins play crucial role in demarcating identity of the two compartments thus ensuring directionality of transport, which is the key to signaling network (Melchior and Gerace, 1998; Vetter et al., 1999). The ubiquitous presence of chromatin assembly/remodeling proteins, for example, histone deacetylase, histone 2A, histone 2B, histone 3, histone 4 was evident. The conspicuous presence of plurifunctional protein 14-3-3 in both monocots and dicot was intriguing, due to its acknowledged role in in ABA signaling, nucleocytoplasmic trafficking and chromatin remodeling, besides its role in development (Brunet et al., 2002). The other prominent functional proteins present were enzymes involved in scavenging of ROS, synthesis of osmolytes and chaperones whose activities enable plants to curtail damage and sustain physiological activity to survive stress conditions.

In a classical proteomics study, Subba et al. (2013) examined and compared the dehydration-responsive nuclear proteome of tolerant and susceptible cultivars of chickpea. The basic stress-responsive features were found to be similar in both the varieties; however, cultivar-specific variations in terms of protein isoforms were observed for several of the common proteins. The tolerant variety was found to maintain better water status and displayed less oxidative damage. These findings highlight a coordinated response of nuclear proteome under dehydration involving both regulatory and functional protein network. Despite a large part of the identified proteins were found to be species-specific, conservation of primary elements is suggestive of a possible analogous mechanism for better adaptation.

## Membrane Proteome Under Water-Deficit

Cell membranes are composed of dynamic lipid–protein matrices constituting the interface between cellular compartments. The lipid component presents a discriminating barrier to solute movement, while the membrane-associated proteins perform distinctive role in metabolism and maintenance of cellular homeostasis during stress adaptation (Jaiswal et al., 2012). Imposition of any form of osmotic stress elicits physical and biochemical signals perceived by membrane proteins at the frontier, which results in torrent of secondary subcellular signal relays. The importance of membrane proteins under abiotic stress has been well recognized and multiple studies have been carried out despite the limitation of their low abundance, hydrophobicity and complex electrophoretic properties (Michelet and Boutry, 1995; Garcia-Gomez et al., 2000; Beffagna et al., 2005; Liu et al., 2005). There have been, however, two noticeable membrane-associated proteomics studies in crop species under osmotic stress (**Table 1**, **Figure 1**). Nouri and Komatsu (2010) developed an osmotic stress responsive proteome and identified 12 and 86 differentially expressed proteins via gel-based and gel-free methods, respectively. Interestingly, proteins belonging to the classes involved in cell structure, metabolism, and protein-folding and protein synthesis were down-regulated under osmotic stress. The proteins involved in transport, signaling or defense were found to be down-regulated. Among the up-regulated proteins, most prominent were the transporters such as H^+^-ATPases, which build membrane potential for energy production, maintain turgor and intracellular pH (**Figure 2**). Another important up-regulated protein was calnexin. Curiously, the transcript abundance of calnexin did not change when compared to unstressed condition, while the immunoblot analysis confirmed the protein abundance in the plasma membrane. This phenomenon is suggestive of possible migration of classical ER-associated signaling protein, calnexin, to plasma membrane under osmotic stress.

Jaiswal et al. (2014) investigated the dehydration-responsive membrane-associated proteome of chickpea by 2-DE coupled with mass spectrometry. Proteomic analysis revealed 184 proteins significantly altered their intensities over dehydration treatments. The DRPs were categorized into 23 classes that were mainly related to generation of precursor metabolites and energy, protein metabolic process, transport and photosynthesis, among others. The dominant DRPs included various units of ATP synthase and light harvesting antenna complexes associated with PSI and PSII, besides oxygen evolving complex. Most of the DRPs associated with these classes were down-regulated, which might cause reduced ROS production, thus preventing cellular damage. The proteins majorly up-regulated were molecular chaperones such as HSPs and BiP. One of the interesting proteins identified was a SUN superfamily nuclear envelope protein. The SUN superfamily proteins are known to ‘bridge’ across the inner and outer nuclear membranes and physically connect the nucleus to every major component of cytoskeleton and serve as both mechanical adaptors and nuclear envelope receptors (Tzur et al., 2006). Functional characterization of CaSUN1 (Jaiswal et al., 2014) confirmed its participation in osmotic stress response, suggesting that SUN superfamily protein might be the signaling connect between cytoplasmic and nucleoplasmic activities for inducing stress response.

## Reprograming of Mitochondrial and Plastid Proteome Under Dehydration

Cellular homeostasis is often disrupted by changes in the extracellular environment that uncouple biochemical pathways and result in undesirable accumulation of ROS. Under osmotic stress, reduction in CO_2_ fixation rate leads to decline in regeneration of NADP^+^ by Calvin cycle. Overreduction of electron transport chain in both plastids and mitochondria forms singlet oxygen (O_2_∗) which impair photosynthesis, creating a metabolic imbalance and generating oxidative distress (Mittler, 2006). Mitochondria and plastid coordinate to limit ROS accumulation and uphold energy balance to ensure cell survival under stress. A comprehensive study by Taylor et al. (2009) combined data from proteomic screening and GFP-targeting analysis to create a list of proteins from these organelles. Data curation yielded 279 non-redundant proteins, of which 5% belonged to peroxisome, 22% to mitochondria and 73% to chloroplast. Mitochondria isolated from stressed pea seedlings maintained their electron transport chain activity, while there was an apparent change in the activity of uncoupling proteins. There was ubiquitous activation of ROS detoxification pathways such as ascorbate/glutathione cycle and SOD-mediated detoxification of O_2_∗. Protection of existing matrix enzymes by synthesis of soluble protein-folding molecular chaperones like HSP22, HSP70, and HSP90 was also observed (**Figure 2**). However, repression of proteins associated with carbon assimilation in chloroplast could be an adjustment of the altered energy levels in plant under osmotic stress.

Chloroplast proteome was investigated following dehydration and subsequent watering in tall grass (*Festuca arundinacea*), between a high and low drought tolerant variety (Kosmala et al., 2012). Under water-deficit conditions, the rate of CO_2_ fixation is reduced, while higher rate of light reaction acts as a source of ROS leading to decrease in photosynthetic efficiency (Mittler, 2006). This phenomenon of photoinhibition results in degradation of proteins such as D1 in PSII. An ATP-dependent zinc FtH metalloprotease was identified, which might be involved in removal of damaged D1 protein from PS II. Another novel class of proteins identified was lipocalins, which had earlier been reported to be associated with chilling stress. These proteins are known to protect the thylakoid membrane from oxidative stress. Further, proteins like fibrillins, which maintain structural integrity of thylakoid membrane, were also detected. Altogether, these proteins were shown to be involved either directly in photosynthetic reactions or in protection of photosynthetic apparatus under stress. Sanda et al. (2011) investigated the effect of water-deficit on photosynthetic electron transport chain and identified 60 proteins that changed in abundance (**Table 1**, **Figure 1**). Most of the proteins identified were either chaperones or proteins related to electron transport chain. Interestingly, the integrity of PSI and PSII could largely be maintained even though carbon fixation rate was subdued.

A significant work by Skirycz et al. (2011), using ^15^N-labeling, established the interdependence of mitochondria and plastid function in *Arabidopsis* subjected to water-deficit condition. This study complemented the transcriptomic data (Skirycz et al., 2011) and provided novel insights into the underlying mechanism of stress responses. The proteome data clearly entailed the cooperative mechanism between chloroplast and mitochondria, which maintains the physiological balance under stress. The dehydration-responsive down-regulated proteins were found to belong mainly to primary metabolism, particularly photosynthesis, photorespiration, glycolysis, TCA cycle, and mitochondrial electron transport chain. On the contrary, the enzymes involved in redox homeostasis such as thioredoxin, APX, and ribosomal proteins were overrepresented.

## Protein Phosphorylation Under Dehydration Stress

Protein phosphorylation is the central post-translational process which co-ordinates the synchronization of stress signals by regulating the protein pool dynamics between the cytosol and rest of the organelles (Pawson and Scott, 2005; Bonhomme et al., 2012). In recent years, the development of sensitive mass spectrometric techniques in conjunction with strategies to enrich the phosphorylated peptides/proteins led to the identification of a large repertoire of phosphoproteins from several plant species (**Table 2**, **Figure 1**). Most efforts in plant phosphoproteomics analyses have focused on the identification of protein phosphorylation in developmental stages (Chitteti and Peng, 2007), cellular compartments (Nuhse, 2004; Ito et al., 2009; Jones et al., 2009), chromatin structure (Tan et al., 2007) and only recently has the focus extended to stress response (Subba et al., 2013; Xue et al., 2013; Stecker et al., 2014; Zhang et al., 2014a,b).

**Table 2:** Number of phosphoproteins identified in different plants under water-deficit conditions.

Plant	Number of proteins	Proteomic method	Reference
*Arabidopsis*	468	Label-free LAXIC	Xue et al., 2013
*Phaseolus vulgaris*	22	2-DE, MS/MS	Yang et al., 2013
Chickpea	91	2-DE, MS/MS	Subba et al., 2013
Wheat	31 (seedling leaves)	LC-MS/MS	Zhang et al., 2014a
Wheat	61 (developed seeds)	LC-MS/MS	Zhang et al., 2014b
*Arabidopsis*	29	^15^N metabolic labeling, MS/MS	Stecker et al., 2014

Xue et al. (2013) introduced a novel mass spectrometry-based label-free quantitation method that facilitated systematic profiling of plant phosphoproteome changes with high efficiency and accuracy. In *Arabidopsis*, 468 up-regulated phosphopeptides representing 497 phosphosites showed significant changes under osmotic stress induced by mannitol and ABA treatment. Several known and novel components in the osmotic stress response pathway were identified. Phosphoproteomic analysis of polyethylene glycol-induced osmotic stress in root tips of common bean (*Phaseolus vulgaris*) was carried out by Yang et al. (2013), which led to the identification of 22 DRPs, of which 10 were found to be phosphorylated. Subba et al. (2013) identified 91 putative phosphoproteins in chickpea under dehydration, presumably involved in a variety of functions including cell defense and rescue, photosynthesis and photorespiration, molecular chaperones, and ion transport. Multiple sites of phosphorylation were predicted on several key elements, which include both the regulatory as well as the functional proteins. A novel protein DREPP (developmentally regulated plasma membrane polypeptide) was found to be differentially regulated under dehydration stress. Two significant studies on wheat developing seeds and leaves were conducted recently by Zhang et al. (2014a,b). The comparative phosphoproteome analysis revealed 63 unique phosphopeptides, corresponding to 61 phosphoproteins in the developing seeds, while 31 proteins showed significant changes in phosphorylation level in the leaves. Functional analysis indicated that some of these proteins might be involved in signal transduction, embryo and endosperm development of grains, and dehydration response and defense under water-deficit conditions. Stecker et al. (2014) identified protein phosphorylation events under osmotic stress using ^15^ N-metabolic labeling and untargeted mass spectrometry. The results indicated that regulatory proteins such as members of the MAPK family are specifically phosphorylated in response to osmotic stress and highlighted the utility of targeted phosphoproteomic analysis in understanding protein regulation networks.

Even though the above-mentioned studies were carried out in distinct species and different tissues using diverse methods of dehydration and varied methods of analysis, three major clusters of phosphorylated proteins were identified ubiquitously. Signaling proteins were the most prominent viz., stress induced MAPK pathway, ABA responsive SnRKs, calcium dependent protein kinases, casein kinase and protein phosphatase 2C, besides others. The second cluster consisted primarily of ribosomal proteins and proteins involved in degradation process, indicating that translation and turnover of proteins is tightly regulated by PTMs (**Figure 2**). Other major class consisted of chaperones, LEA proteins and dehydrin, among others involved in protecting the cellular components. These data are particularly important, at least in part, owing to the fact that the currently available stress-responsive phosphoproteins in plant are under-represented. Subcellular phosphoproteomics studies, under stress, are future imperative for understanding in detail the crosstalk between organelles orchestrated by PTM events, which cannot be elucidated by genomic investigations.

## Dehydration-Responsive Proteome Network

Exposure of plants to water-deficit conditions leads to wide range of changes in protein expression levels. Protein composition of a cell is representative of multiple inflections such as protein sorting, translocation, post-translational modification, and protein degradation, all of which are influenced by stress conditions. More importantly, the distribution of proteins in various organelles under stress and their crosstalk to regulate stress responses is the key to understanding cellular mechanisms, which may not be accurately predicted based on genome expression profiling. In this review, the proteome changes in major organelles under osmotic stress have been re-visited, which can be summarized under three adaptive mechanisms: stress perception and signaling, defense response and metabolic regulation and detailed as follows:

### Stress Perception And Signaling

**FIGURE 3:**
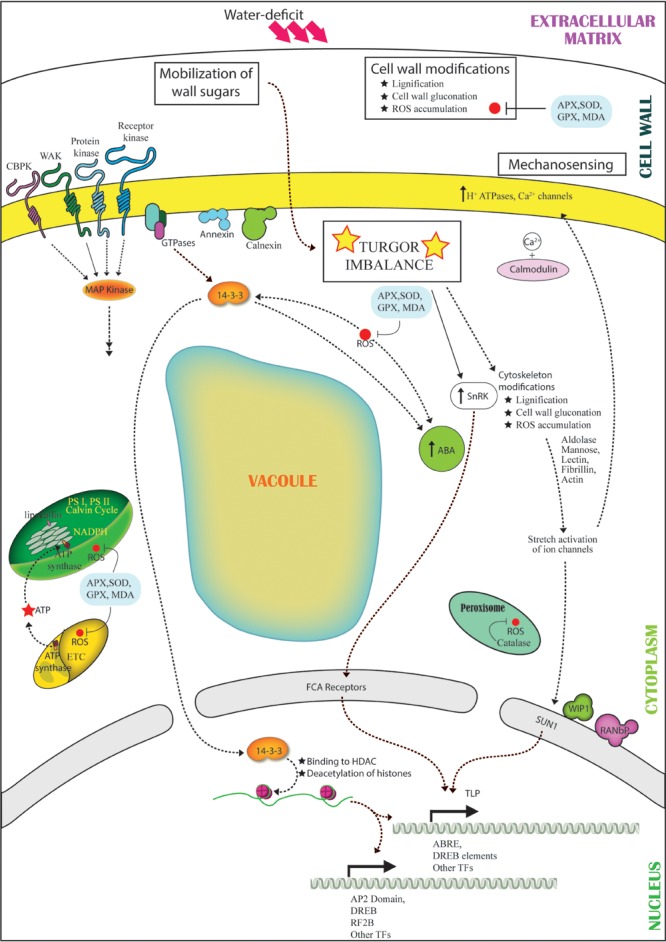
**Representation of the cross talk among different pathways under water-deficit conditions.** Dehydration stress leads to turgor imbalance and production of ROS. The turgor imbalance is mechanosensed, leading to modification in cytoskeleton proteins (lectins, actin, fibrillin) and sugars (mannose, aldolase) in turn leading to stretch-activation of ion channels (H^+^ ATPases, Ca^2+^) and activation of Ca^2+^/calmodulin signaling pathway. Wall sugars are mobilized and activate SnRK. Cell wall undergoes modifications like lignification and contraction. The stress is sensed by receptors (CBPK, WAKs, protein kinases, receptor kinases), which interact with GTPase. GTPase activates ABA and 14-3-3. Level of 14-3-3 increases with ROS production and parallely interacts and activates ABA and SnRK. 14-3-3 translocates to the nucleus and interacts with ACDH leading to choromatin remodeling and activation of transcription factors (AP2, DREB, RF2B, TLP). ABA interacts with the FCA receptors, and nuclear envelope protein SUN1 detects the ion channel activation owing to mechanosensing further leading to activation of TFs.

Perception of osmotic stress, as it emerged from the analysis, is a complex phenomenon that comprises multitude of signaling pathways. The primary inducer of all stress-sensing mechanisms is any kind of change in intracellular osmotic balance. Alteration in cell turgor pressure leads to activation of proteins in the cell membrane and extracellular matrix viz., WAKs and receptor kinases, which may interact with signaling proteins specifically 14-3-3, MAP kinases and protein kinases in the cytosol. The interactions relay the signal to activate several families of transcription factors in the nucleus including WRKY, AP2, DREB, EREBP, RF2B, and leucine zipper. These cues also activate Ca^2+^ channels and ROS secondary signaling pathways, which further exemplify the stress signal via CDPKs to ensue modulation of stress-responsive components such as annexins, calnexins, and calmodulins. Annexin and calnexin are known to translocate to the cell membrane under dehydration, which enhances association with other molecules in the membrane, both resulting in the activation of the downstream signaling cascade (Lee et al., 2006; Jia et al., 2009). Both the proteins are also essential part of ABA-mediated signaling pathway. The reactive oxygen moieties induce the ABA-mediated pathways by activation of signaling proteins like SnRKs, protein phosphatase 2C and intermediates like 14-3-3 proteins in the cell membrane and cytosol, FCA receptor in the nuclear membrane and transcription factor such as tubby like protein (**Figure 3**). Proteins involved in nucleocytoplasmic transport such as RAN, WIP1, dyamin and RANbP ensure the directionality of signal relays and facilitate the overall signaling network.

Cellular mechanosensing of osmotic imbalance may also be a potential mode of perception of stress acuity. The stretch activation of cytoskeleton associated proteins due to changes in the osmotic pressure and ion imbalance created by dehydration may lead to physical signal relays to modulate regulatory nuclear proteins. Further, identification of LINC (linker of nucleoskeleton and cytoskeleton) complex members such as SUN-domain protein, WIP1 and RANbP in the nucleus reinforce the theory of dehydration-induced mechanotransmission (**Figure 3**).

### Defense Response

The mechanisms of dehydration avoidance vs. dehydration tolerance form the basis for understanding and interpreting dehydration-responsive events. The primary response of plants under water-deficit is to avoid further loss of water. The proton pumps associated with the plasma membrane facilitate stomatal closure under dehydration by mediating the eﬄux of K^+^ and Ca^2+^, which regulates the activity of plasma membrane H^+^-ATPase to maintain homeostasis of intracellular ions (Komatsu et al., 2009). Furthermore, lignification of cell wall is a preventive measure, which aids in preserving the intracellular moisture content and ion balance. Multiple stress-responsive proteins such as Ado-met, methyl transferase, AdoHcyase, adenosine kinase are known to be a part of lignification pathway. An alternate mechanism to maintain turgor under dehydration is the breakdown of cell wall polysaccharides by glycosyl hydrolases such as beta galactosidase, xyloglucan hydrolase and hexosaminidase, identified in the ECM, to direct sugars to cytoplasm (Bhushan et al., 2007; Pandey et al., 2010). Additionally, up-regulated proteins such as Δ1-pyrroline-5-carboxylate synthase, involved in biosynthesis of osmoprotectants and sugar metabolizing enzymes like sucrose synthase were also evident (**Figures 2** and **3**).

The production of ROS under dehydration can act as signaling molecules for stress response, although overproduction of ROS results in cellular damage. The survival of plant largely depends on the ROS accumulation under stress against the detoxification process mediated by ROS-scavenging proteins (**Figures 2** and **3**). As evident from proteome data, the electron transport chain in the chloroplast is repressed to avoid oxidative damage to the cell. Reduced expression of oxygen evolving complex proteins, PSI and PSII associated proteins might also enhance cell defense. Furthermore, accumulation of ROS-scavenger proteins such as SOD, ascorbate peroxidase, catalase and glutathione peroxidase under dehydration was noticed in all subcellular compartments. Proteins like lipocalin and fibrillins in the chloroplast were observed to be overexpressed under dehydration and are known to protect thylakoid membranes and PSII, respectively, against photoinhibition induced oxidative stress (Levesque-Tremblay et al., 2009; Singh et al., 2011).

Disturbance in the intracellular pH due to change in solute concentration under dehydration has an adverse effect on protein folding. Accumulation of molecular chaperons such as HSPs and Bip helps in protecting the existing proteins via folding and refolding of the misfolded proteins. Accumulation of RNA-binding proteins, dehydrins and LEA proteins could be additional support for protection of cellular translational apparatus. Furthermore, misfolded proteins, unassembled subunits of multimeric proteins and/or mutated proteins are degraded via intracellular proteolysis, particularly in the cytosol and the nucleus as revealed by phosphoproteome analysis (**Figure 2**).

### Metabolic Regulations

When subjected to water-deficit conditions, plants dynamically alter growth rates and redistribute resources for better survival. Growth reduction increases the survival rate under severe stress but under moderate stress, it can be counterproductive. The proteome data establishes that primary metabolism pathways such as plastidial ATPase, Calvin cycle, and photorespiration are down-regulated, but mitochondrial ATP synthesis is up-regulated, indicating the importance of mitochondria in assisting plastid functions during water-deficit conditions (Skirycz et al., 2011). Under stress, plants try to achieve a balance by preserving the integrity of PSI and PSII, while compromising on overall carbon fixation rates (Sanda et al., 2011). The role of mitochondria in supporting plastid function by recycling reducing equivalents and redirecting its ATP pool is a classic example of optimization of resources under stress. This altered metabolic dynamics among the organelles under stress, emphasizes the importance of proteome-based approaches for systematic understanding of biological processes.

## Concluding Remarks

Screening of organellar proteomes under water-deficit conditions revealed new and interesting insights into modulations of stress response in plants (**Figure 3**). The number of organellar proteomics studies available to date is limited and much work is required to enrich the database. Studies on cell membrane are important to fill the missing links in the signaling pathway. Besides, studies on fractionated cytosolic proteomes are rare and should be taken up to build the remaining chain of events. Moreover, cross-species comparisons for all the compartments and tissue-specific studies are required to postulate a comprehensive mechanism of dehydration progression and the plant responses. We, however, hope that this summary will not only be beneficial in understanding molecular basis of acquisition of dehydration tolerance at the organellar level, but will also highlight the importance of studying changes in compartment-specific protein abundance.

## Author Contributions

NC conceived the study. DG, YR, and SG collated the data. DG, SC, and NC discussed the study and wrote the article.

## Conflict of Interest Statement

The authors declare that the research was conducted in the absence of any commercial or financial relationships that could be construed as a potential conflict of interest.

## Abbreviations

2-DE: two-dimensional electrophoresis
ABA: abscisic acid
APX: ascorbate peroxidise
ATP: adenosine triphosphate
DRNPs: dehydration-responsive nuclear proteome
DRPs: dehydration-responsive proteins
ECM: extra cellular matrix
GFP: green florescent protein
GPX: glutathione peroxidise
GTPase: guanosine triphosphatase
H_2_O_2_: hydrogen peroxide
HSP: heat shock protein
iTRAQ: isobaric tag for relative and absolute quantification
LEA: late embryogenesis abundance
MAPK: mitogen activated protein kinase
MDA: malondialdehyde
NADP: nicotinamide adenine dinucleotide phosphate
NDK: nucleoside diphosphate kinase
PSI & PSII: photosystem I & II
PTMs: post-translational modifications
RAN: ras-related nuclear protein
ROS: reactive oxygen species
RWC: relative water content
SOD: superoxide dismutase
TCA: tricarboxylic acid
WAKs: wall associated kinases

## References

[B1] Abdalla K. O. Baker B. Rafudeen M. S. (2010). Proteomic analysis of nuclear proteins during dehydration of the resurrection plant *Xerophyta viscosa*. *Plant Growth Regul.* 62 279–292. 10.1007/s10725-010-9497-2

[B2] Abdalla K. O. Rafudeen M. S. (2012). Analysis of the nuclear proteome of the resurrection plant *Xerophyta viscosa* in response to dehydration stress using iTRAQ with 2DLC and tandem mass spectrometry. *J. Proteomics* 75 2361–2374. 10.1016/j.jprot.2012.02.006 22361341

[B3] Anderson C. M. Wagner T. A. Perret M. He Z. H. He D. Kohorn B. D. (2001). WAKs: cell wall-associated kinases linking the cytoplasm to the extracellular matrix. *Plant Mol. Biol.* 47 197–206. 10.1023/A:1010691701578 11554472

[B4] Apel K. Hirt H. (2004). REACTIVE OXYGEN SPECIES: metabolism. Oxidative Stress and Signal Transduction. *Annu. Rev. Plant Biol.* 55 373–399. 10.1146/annurev.arplant.55.031903.141701 15377225

[B5] Bae M. S. Cho E. J. Choi E.-Y. Park O. K. (2003). Analysis of the *Arabidopsis* nuclear proteome and its response to cold stress. *Plant J.* 36 652–663. 10.1046/j.1365-313X.2003.01907.x 14617066

[B6] Bartels D. Sunkar R. (2005). Drought and salt tolerance in plants. *Crit. Rev. Plant Sci.* 24 23–58. 10.1080/07352680590910410

[B7] Beffagna N. Buffoli B. Busi C. (2005). Modulation of reactive oxygen species production during osmotic stress in *Arabidopsis thaliana* cultured cells: involvement of the plasma membrane Ca^2+^-ATPase and H^+^-ATPase. *Plant Cell Physiol.* 46 1326–1339. 10.1093/pcp/pci142 15937326

[B8] Bhushan D. Jaiswal D. K. Ray D. Basu D. Datta A. Chakraborty S. (2011). Dehydration-responsive reversible and irreversible changes in the extracellular matrix: comparative proteomics of chickpea genotypes with contrasting tolerance. *J. Proteome Res.* 10 2027–2046. 10.1021/pr200010f 21348435

[B9] Bhushan D. Pandey A. Chattopadhyay A. Choudhary M. K. Chakraborty S. Datta A. (2006). Extracellular matrix proteome of chickpea (*Cicer arietinum* L.) illustrates pathway abundance, novel protein functions and evolutionary perspect. *J. Proteome Res.* 5 1711–1720. 10.1021/pr060116f 16823979

[B10] Bhushan D. Pandey A. Choudhary M. K. Datta A. Chakraborty S. Chakraborty N. (2007). Comparative proteomics analysis of differentially expressed proteins in chickpea extracellular matrix during dehydration stress. *Mol. Cell Proteomics* 6 1868–1884. 10.1074/mcp.M700015-MCP200 17686759

[B11] Bohnert H. J. Gong Q. Li P. Ma S. (2006). Unraveling abiotic stress tolerance mechanisms-getting genomics going. *Curr. Opin. Plant Biol.* 9 180–188. 10.1016/j.pbi.2006.01.003 16458043

[B12] Bonhomme L. Valot B. Tardieu F. Zivy M. (2012). Phosphoproteome dynamics upon changes in plant water status reveal early events associated with rapid growth adjustment in maize leaves. *Mol. Cell Proteomics* 11 957–972. 10.1074/mcp.M111.015867 22787273 PMC3494150

[B13] Boyer J. S. (1982). Plant productivity and environment. *Science* 218 443–448. 10.1126/science.218.4571.443 17808529

[B14] Brunet A. Kanai F. Stehn J. Xu J. Sarbassova D. Frangioni J. V. (2002). 14-3-3 transits to the nucleus and participates in dynamic nucleocytoplasmic transport. *J. Cell Biol.* 156 817–828. 10.1083/jcb.200112059 11864996 PMC2173313

[B15] Buhr N. Carapito C. Schaeffer C. Kieffer E. Van Dorsselaer A. Viville S. (2008). Nuclear proteome analysis of undifferentiated mouse embryonic stem and germ cells. *Electrophoresis* 29 2381–2390. 10.1002/elps.200700738 18449859

[B16] Carpita N. C. Gibeaut D. M. (1993). Structural models of primary cell walls in flowering plants: consistency of molecular structure with the physical properties of the walls during growth. *Plant J.* 3 1–30. 10.1111/j.1365-313X.1993.tb00007.x 8401598

[B17] Chen J. Zhang Y. Wang C. Lü W. Jin J. B. Hua X. (2011). Proline induces calcium-mediated oxidative burst and salicylic acid signaling. *Amino Acids* 40 1473–1484. 10.1007/s00726-010-0757-2 20890619

[B18] Chitteti B. R. Peng Z. (2007). Proteome and phosphoproteome dynamic change during cell dedifferentiation in *Arabidopsis*. *Proteomics* 7 1473–1500. 10.1002/pmic.200600871 17407188

[B19] Choudhary M. K. Basu D. Datta A. Chakraborty N. Chakraborty S. (2009). Dehydration-responsive nuclear proteome of rice (*Oryza sativa* L.) illustrates protein network, novel regulators of cellular adaptation, and evolutionary perspective. *Mol. Cell Proteomics* 8 1579–1598. 10.1074/mcp.M800601-MCP200 19321431 PMC2709188

[B20] Cooper B. Campbell K. B. Feng J. Garrett W. M. Frederick R. (2011). Nuclear proteomic changes linked to soybean rust resistance. *Mol. Biosyst.* 7 773–783. 10.1039/c0mb00171f 21132161

[B21] Elmayan T. Fromentin J. Riondet C. Alcaraz G. Blein J.-P. Simon-Plas F. (2007). Regulation of reactive oxygen species production by a 14-3-3 protein in elicited tobacco cells. *Plant Cell Environ.* 30 722–732. 10.1111/j.1365-3040.2007.01660.x 17470148

[B22] Fukamatsu Y. Yabe N. Hasunuma K. (2003). *Arabidopsis* NDK1 is a component of ROS signaling by interacting with three catalases. *Plant Cell Physiol.* 44 982–989. 10.1093/pcp/pcg140 14581623

[B23] Garaeva L. D. Pozdeeva S. A. Timofeeva O. A. Khokhlova L. P. (2006). Cell-wall lectins during winter wheat cold hardening. *Russ. J. Plant Physiol.* 53 746–750. 10.1134/S1021443706060033

[B24] Garcia-Gomez B. I. Campos F. Hernandez M. Covarrubias A. A. (2000). Two bean cell wall proteins more abundant during water deficit are high in proline and interact with a plasma membrane protein. *Plant J.* 22 277–288. 10.1046/j.1365-313x.2000.00739.x 10849345

[B25] Golldack D. Li C. Mohan H. Probst N. (2014). Tolerance to drought and salt stress in plants: unraveling the signaling networks. *Front. Plant Sci.* 5:151 10.3389/fpls.2014.00151 PMC4001066 24795738

[B26] Gygi S. P. Rochon Y. Franza B. R. Aebersold R. (1999). Correlation between protein and mRNA abundance in yeast. *Mol. Cell. Biol.* 19 1720–1730. 10.1128/MCB.19.3.1720 10022859 PMC83965

[B27] Henrich S. Cordwell S. J. Crossett B. Baker M. S. Christopherson R. I. (2007). The nuclear proteome and DNA-binding fraction of human Raji lymphoma cells. *Biochim. Biophys. Acta* 1774 413–432. 10.1016/j.bbapap.2006.12.011 17369005

[B28] Hossain Z. Komatsu S. (2013). Contribution of proteomic studies towards understanding plant heavy metal stress response. *Front. Plant Sci.* 3:310 10.3389/fpls.2012.00310 PMC3555118 23355841

[B29] Ito J. Taylor N. L. Castleden I. Weckwerth W. Millar A. H. Heazlewood J. L. (2009). A survey of the *Arabidopsis thaliana* mitochondrial phosphoproteome. *Proteomics* 9 4229–4240. 10.1002/pmic.200900064 19688752

[B30] Jaiswal D. K. Mishra P. Subba P. Rathi D. Chakraborty S. Chakraborty N. (2014). Membrane-associated proteomics of chickpea identifies Sad1/UNC-84 protein (CaSUN1), a novel component of dehydration signaling. *Sci. Rep.* 4:4177 10.1038/srep04177 PMC3937784 24577507

[B31] Jaiswal D. K. Ray D. Subba P. Mishra P. Gayali S. Datta A. (2012). Proteomic analysis reveals the diversity and complexity of membrane proteins in chickpea (*Cicer arietinum* L.). *Proteome Sci.* 10:59 10.1186/1477-5956-10-59 PMC3558352 23031650

[B32] Jewett T. J. Sibley L. D. (2003). Aldolase forms a bridge between cell surface adhesins and the actin cytoskeleton in apicomplexan parasites. *Mol. Cell* 11 885–894. 10.1016/S1097-2765(03)00113-8 12718875

[B33] Jia X. Y. He L. H. Jing R. L. Li R. Z. (2009). Calreticulin: Conserved protein and diverse functions in plants. *Physiol. Plant.* 136 127–138. 10.1111/j.1399-3054.2009.1223.x 19453510

[B34] Jones A. M. E. MacLean D. Studholme D. J. Serna-Sanz A. Andreasson E. Rathjen J. P. (2009). Phosphoproteomic analysis of nuclei-enriched fractions from *Arabidopsis thaliana*. *J. Proteomics* 72 439–451. 10.1016/j.jprot.2009.02.004 19245862

[B35] Komatsu S. Wada T. Abaléa Y. Nouri M.-Z. Nanjo Y. Nakayama N. (2009). Analysis of plasma membrane proteome in soybean and application to flooding stress response. *J. Prot. Res.* 8 4487–4499. 10.1021/pr9002883 19658398

[B36] Kosmala A. Perlikowski D. Pawlowicz I. Rapacz M. (2012). Changes in the chloroplast proteome following water deficit and subsequent watering in a high- and a low-drought-tolerant genotype of *Festuca arundinacea*. *J. Exp. Bot.* 63 6161–6172. 10.1093/jxb/ers265 23045610

[B37] Lata C. Bhutty S. Bahadur R. P. Majee M. Prasad M. (2011). Association of an SNP in a novel DREB2-like gene SiDREB2 with stress tolerance in foxtail millet [*Setaria italica* (L.)]. *J. Exp. Bot.* 62 3387–3401. 10.1093/jxb/err016 21414959 PMC3130168

[B38] Lee B. J. Kwon S. J. Kim S.-K. Kim K.-J. Park C.-J. Kim Y.-J. (2006). Functional study of hot pepper 26S proteasome subunit RPN7 induced by *Tobacco mosaic* virus from nuclear proteome analysis. *Biochem. Biophys. Res. Commun.* 351 405–411. 10.1016/j.bbrc.2006.10.071 17070775

[B39] Levesque-Tremblay G. Havaux M. Ouellet F. (2009). The chloroplastic lipocalin AtCHL prevents peroxidation and protects *Arabidopsis* against oxidative stress. *Plant J.* 60 691–702. 10.1111/j.1365-313X.2009.03991.x 19674405

[B40] Liu H. Wang M. Chou K.-C. (2005). Low-frequency Fourier spectrum for predicting membrane protein types. *Biochem. Biophys. Res. Commun.* 336 737–739. 10.1016/j.bbrc.2005.08.160 16140260

[B41] Melchior F. Gerace L. (1998). Two-way trafficking with Ran. *Trends Cell Biol.* 8 175–179. 10.1016/S0962-8924(98)01252-5 9695834

[B42] Michelet B. Boutry M. (1995). The plasma membrane H^+^ -ATPase (A highly regulated enzyme with multiple physiological functions). *Plant Physiol.* 108 1–6. 12228449 10.1104/pp.108.1.1 PMC157299

[B43] Mittler R. (2006). Abiotic stress, the field environment and stress combination. *Trends Plant Sci.* 11 15–19. 10.1016/j.tplants.2005.11.002 16359910

[B44] Moschou P. N. Paschalidis K. A. Delis I. D. Andriopoulou A. H. Lagiotis G. D. Yakoumakis D. I. (2008). Spermidine exodus and oxidation in the apoplast induced by abiotic stress is responsible for H_2_O_2_ signatures that direct tolerance responses in tobacco. *Plant Cell* 20 1708–1724. 10.1105/tpc.108.059733 18577660 PMC2483379

[B45] Narula K. Datta A. Chakraborty N. Chakraborty S. (2013). Comparative analyses of nuclear proteome: extending its function. *Front. Plant Sci.* 4:100 10.3389/fpls.2013.00100 PMC3636469 23637696

[B46] Nouri M.-Z. Komatsu S. (2010). Comparative analysis of soybean plasma membrane proteins under osmotic stress using gel-based and LC MS/MS-based proteomics approaches. *Proteomics* 10 1930–1945. 10.1002/pmic.200900632 20209511

[B47] Nuhse T. S. (2004). Phosphoproteomics of the *Arabidopsis* plasma membrane and a new phosphorylation site database. *Plant Cell* 16 2394–2405. 10.1105/tpc.104.023150 15308754 PMC520941

[B48] Pandey A. Chakraborty S. Datta A. Chakraborty N. (2008). Proteomics approach to identify dehydration responsive nuclear proteins from chickpea (*Cicer arietinum* L.). *Mol. Cell Proteomics* 7 88–107. 10.1074/mcp.M700314-MCP200 17921517

[B49] Pandey A. Rajamani U. Verma J. Subba P. Chakraborty N. Datta A. (2010). Identification of extracellular matrix proteins of rice (*Oryza sativa* L.) involved in dehydration-responsive network: a proteomic approach. *J. Proteome Res.* 9 3443–3464. 10.1021/pr901098p 20433195

[B50] Papadakis A. K. Roubelakis-Angelakis K. A. (2005). Polyamines inhibit NADPH oxidase-mediated superoxide generation and putrescine prevents programmed cell death induced by polyamine oxidase-generated hydrogen peroxide. *Planta* 220 826–837. 10.1007/s00425-004-1400-9 15517351

[B51] Pawson T. Scott J. D. (2005). Protein phosphorylation in signaling-50 years and counting. *Trends Biochem. Sci.* 30 286–290. 10.1016/j.tibs.2005.04.013 15950870

[B52] Pechanova O. Hsu C.-Y. Adams J. P. Pechan T. Vandervelde L. Drnevich J. (2010). Apoplast proteome reveals that extracellular matrix contributes to multistress response in poplar. *BMC Genomics* 11:674 10.1186/1471-2164-11-674 PMC3091788 21114852

[B53] Poschet G. Hannich B. Raab S. Jungkunz I. Klemens P. A. W. Krueger S. (2011). A novel *Arabidopsis* vacuolar glucose exporter is involved in cellular sugar homeostasis and affects the composition of seed storage compounds. *Plant Physiol.* 157 1664–1676. 10.1104/pp.111.186825 21984725 PMC3327193

[B54] Repetto O. Rogniaux H. Firnhaber C. Zuber H. Küster H. Larré C. (2008). Exploring the nuclear proteome of *Medicago truncatula* at the switch towards seed filling. *Plant J.* 56 398–410. 10.1111/j.1365-313X.2008.03610.x 18643982

[B55] Repetto O. Rogniaux H. Larré C. Thompson R. Gallardo K. (2012). The seed nuclear proteome. *Front. Plant Sci.* 3:289 10.3389/fpls.2012.00289 PMC3526781 23267364

[B56] Salzano A. M. Paron I. Pines A. Bachi A. Talamo F. Bivi N. (2006). Differential proteomic analysis of nuclear extracts from thyroid cell lines. *J. Chromatogr. B Analyt. Technol. Biomed. Life Sci.* 833 41–50. 10.1016/j.jchromb.2005.12.025 16431169

[B57] Sanda S. Yoshida K. Kuwano M. Kawamura T. Munekage Y. N. Akashi K. (2011). Responses of the photosynthetic electron transport system to excess light energy caused by water deficit in wild watermelon. *Physiol. Plant.* 142 247–264. 10.1111/j.1399-3054.2011.01473.x 21438881

[B58] Seki M. Narusaka M. Ishida J. Nanjo T. Fujita M. Oono Y. (2002). Monitoring the expression profiles of 7000 *Arabidopsis* genes under drought, cold and high-salinity stresses using a full-length cDNA microarray. *Plant J.* 31 279–292. 10.1046/j.1365-313X.2002.01359.x 12164808

[B59] Shinozaki K. Yamaguchi-Shinozaki K. Seki M. (2003). Regulatory network of gene expression in the drought and cold stress responses. *Curr. Opin. Plant Biol.* 6 410–417. 10.1016/S1369-5266(03)00092-X 12972040

[B60] Singh R. K. Anandhan S. Singh S. Patade V. Y. Ahmed Z. Pande V. (2011). Metallothionein-like from *Cicer microphyllumis* regulated by multiple abiotic stresses. *Protoplasma* 248 839–847. 10.1007/s00709-010-0249-y 21161305

[B61] Skirycz A. Memmi S. De Bodt S. Maleux K. Obata T. Fernie A. R. (2011). A reciprocal 15 N-labeling proteomic analysis of expanding *Arabidopsis* leaves subjected to osmotic stress indicates importance of mitochondria in preserving plastid functions. *J. Proteome Res.* 10 1018–1029. 10.1021/pr100785n 21142212

[B62] Stecker K. E. Minkoff B. B. Sussman M. R. (2014). Phosphoproteomic analyses reveal early signaling events in the osmotic stress response. *Plant Physiol.* 165 1171–1187. 10.1104/pp.114.238816 24808101 PMC4081330

[B63] Subba P. Kumar R. Gayali S. Shekhar S. Parveen S. Pandey A. (2013). Characterisation of the nuclear proteome of a dehydration-sensitive cultivar of chickpea and comparative proteomic analysis with a tolerant cultivar. *Proteomics* 13 1973–1992. 10.1002/pmic.201200380 23798506

[B64] Tan F. Li G. Chitteti B. R. Peng Z. (2007). Proteome and phosphoproteome analysis of chromatin associated proteins in rice (*Oryza sativa*). *Proteomics* 7 4511–4527. 10.1002/pmic.200700580 18022940

[B65] Taylor N. L. Tan Y.-F. Jacoby R. P. Millar A. H. (2009). Abiotic environmental stress induced changes in the *Arabidopsis thaliana* chloroplast, mitochondria and peroxisome proteomes. *J. Proteomics* 72 367–378. 10.1016/j.jprot.2008.11.006 19061979

[B66] Tzur Y. B. Wilson K. L. Gruenbaum Y. (2006). SUN-domain proteins: “Velcro” that links the nucleoskeleton to the cytoskeleton. *Nat. Rev. Mol. Cell Biol.* 7 782–788. 10.1038/nrm2003 16926857

[B67] Varma P. Mishra R. K. (2011). Dynamics of nuclear matrix proteome during embryonic development in *Drosophila melanogaster*. *J. Biosci.* 36 439–459. 10.1007/s12038-011-9081-6 21799256

[B68] Vera-Estrella R. (2005). Salt stress in *Thellungiella halophila* activates Na^+^ transport mechanisms required for salinity tolerance. *Plant Physiol.* 139 1507–1517. 10.1104/pp.105.067850 16244148 PMC1283785

[B69] Verma J. K. Gayali S. Dass S. Kumar A. Parveen S. Chakraborty S. (2014). OsAlba1, a dehydration-responsive nuclear protein of rice (*Oryza sativa* L. ssp. indica), participates in stress adaptation. *Phytochemistry* 100 16–25. 10.1016/j.phytochem.2014.01.015 24534105

[B70] Verslues P. E. Agarwal M. Katiyar-Agarwal S. Zhu J. Zhu J.-K. (2006). Methods and concepts in quantifying resistance to drought, salt and freezing, abiotic stresses that affect plant water status. *Plant J.* 45 523–539. 10.1111/j.1365-313X.2005.02593.x 16441347

[B71] Vetter I. R. Nowak C. Nishimoto T. Kuhlmann J. Wittinghofer A. (1999). Structure of a Ran-binding domain complexed with Ran bound to a GTP analogue: implications for nuclear transport. *Nature* 398 39–46. 10.1038/17969 10078529

[B72] Wu X. Shiroto Y. Kishitani S. Ito Y. Toriyama K. (2009). Enhanced heat and drought tolerance in transgenic rice seedlings overexpressing OsWRKY11 under the control of HSP101 promoter. *Plant Cell Rep.* 28 21–30. 10.1007/s00299-008-0614-x 18818929

[B73] Xue L. Wang P. Wang L. Renzi E. Radivojac P. Tang H. (2013). Quantitative measurement of phosphoproteome response to osmotic stress in *Arabidopsis* based on Library-Assisted eXtracted Ion Chromatogram (LAXIC). *Mol. Cell Proteomics* 12 2354–2369. 10.1074/mcp.O113.027284 23660473 PMC3734591

[B74] Yang Z.-B. Eticha D. Führs H. Heintz D. Ayoub D. Van Dorsselaer A. (2013). Proteomic and phosphoproteomic analysis of polyethylene glycol-induced osmotic stress in root tips of common bean (*Phaseolus vulgaris* L.). *J. Exp. Bot.* 64 5569–5586. 10.1093/jxb/ert328 24123251 PMC3871817

[B75] Zhang M. Lv D. Ge P. Bian Y. Chen G. Zhu G. (2014a). Phosphoproteome analysis reveals new drought response and defense mechanisms of seedling leaves in bread wheat (*Triticum aestivum* L.). *J. Proteomics* 109 290–308. 10.1016/j.jprot.2014.07.010 25065648

[B76] Zhang M. Ma C.-Y. Lv D.-W. Zhen S.-M. Li X.-H. Yan Y.-M. (2014b). Comparative phosphoproteome analysis of the developing grains in bread wheat (*Triticum aestivum* L.) under well-watered and water-deficit conditions. *J. Proteome Res.* 13 4281–4297. 10.1021/pr500400t 25145454

[B77] Zhu J. Alvarez S. Marsh E. L. Lenoble M. E. Cho I.-J. Sivaguru M. (2007). Cell wall proteome in the maize primary root elongation zone. II. Region-specific changes in water soluble and lightly ionically bound proteins under water deficit. *Plant Physiol.* 145 1533–1548. 10.1104/pp.107.107250 17951457 PMC2151692

